# Breeding Practice Improves the Mycorrhizal Responsiveness of Cotton (*Gossypium* spp. L.)

**DOI:** 10.3389/fpls.2021.780454

**Published:** 2021-12-10

**Authors:** Letian Wang, Xihe Wang, Baidengsha Maimaitiaili, Arjun Kafle, Khuram Shehzad Khan, Gu Feng

**Affiliations:** ^1^College of Resources and Environmental Sciences, National Academy of Agriculture Green Development, China Agricultural University, Beijing, China; ^2^Institute of Soil and Fertilizer and Agricultural Sparing Water, Xinjiang Academy of Agricultural Science, Urumqi, China; ^3^Institute of Nuclear Technology and Biotechnology, Xinjiang Academy of Agricultural Sciences, Urumqi, China; ^4^Department of Crop and Soil Sciences, North Carolina State University, Raleigh, NC, United States

**Keywords:** cotton cultivars, breeding, mycorrhizal responsiveness, indigenous community, phosphorus uptake

## Abstract

Maximizing the function of indigenous arbuscular mycorrhizal (AM) fungi by choosing specific crop genotypes offers one of the few untapped opportunities to improve the sustainability of agriculture. In this study, the differences in mycorrhizal responsiveness (MR) in plant growth and shoot phosphorus (P) content among cotton (*Gossypium* spp. L.) genotypes from different release dates were compared and then the relationships between MR and P uptake-related traits were determined. The experimental design in a greenhouse included 24 genotypes released from 1950 to present in Xinjiang Province, inoculation with or without AM fungi, and P levels (15 and 150 mg P kg^–1^ added as KH_2_PO_4_). Results showed that the modern cotton genotypes exhibited a higher degree of mycorrhizal colonization, the hyphal length density (HLD), and mycorrhizae-induced changes in shoot growth than the old genotypes when inoculated with indigenous AM fungi at both the P levels. Moreover, MR was highly correlated with the HLD at low P levels and the HLD may provide useful insights for future cotton breeding aimed at delivering crop genotypes that can benefit more from AM fungi.

## Introduction

Agricultural intensification has led to an increased yield of staple crops (Tilman, 1999; Godfray et al., 2010), but an intensive supply of chemical fertilizers has brought a series of adverse environmental impacts and sustainability issues (Foley et al., 2005; Bender et al., 2016). Arbuscular mycorrhizal (AM) fungi are widely distributed in natural and agricultural ecosystems and the most recognized benefit of AM fungi is enhanced plant uptake of immobile nutrients, especially phosphorus (P). Exploitation of the function of AM fungi to increase plant nutrient uptake offers one of the few untapped opportunities to reduce P fertilizer application and improve the sustainability of agriculture (Toju et al., 2018; Zhang et al., 2019). Whether or not inoculated, AM fungi can successfully establish and enhance plant growth that depends on a series of factors such as native AM fungal communities, pesticides, and cultivars (Bethlenfalvay et al., 1996; Busse et al., 2004; Muok et al., 2009). Hence, this approach is not always stable and may even have a neutral effect on crop production (Jeffries et al., 2003; Corkidi et al., 2004; Lekberg et al., 2008; Cely et al., 2016). Besides inoculating nonindigenous AM fungi, using the indigenous AM fungal community to enhance plant growth could be an alternative. P availability is considered to be the major driving force of the mycorrhizal responsiveness (MR) of plants (Johnson et al., 2010). High soil P availability causes a decrease in AM fungal colonization and mycorrhizal benefits (Mader et al., 2000). However, a rich community of AM fungi in agricultural ecosystems has been observed with intensive crop management (Oehl et al., 2004), regardless of the application of fertilizers, pesticides, and even high soil available P (Thomson et al., 1992; Kaeppler et al., 2000; Hao et al., 2008; Gai et al., 2010; Vestberg et al., 2011). Moreover, the indigenous AM fungal communities can play a role in promoting growth of maize and cotton (Liu et al., 2016). According to breeding practices and release date, a recent study divided maize varieties into three varietal groups, including old landraces, modern hybrids, and inbred lines, and compared MR of these varietal groups (Wang et al., 2020b). The results showed that there were no significant differences among varietal groups for MR, confirming that the ability to associate with and benefit from AM fungi has been maintained in modern crops (Wang et al., 2020b). In addition, the varietal group of modern hybrids inoculated with indigenous AM fungi exhibited the higher P acquisition and higher P use efficiency under field conditions (Wang et al., 2020b). Thus, maximizing the benefits of indigenous AM fungi could be a useful way to enhance crop growth, thereby reducing the dependence of crop production on the addition of P chemical fertilizers.

Mycorrhizal responsiveness reflects the degree of the response of plants to AM colonization and the capacity to benefit from the symbiosis (Janos, 2007; Sawers et al., 2010). MR is determined by the complexity of combinations of plant-funga environmental conditions, and the magnitude of MR exhibites great variation (Yao et al., 2001a,b; Hao et al., 2008; Berger and Gutjahr, 2021). Specifically, MR depends on root morphological, physiological, and mycorrhizal traits that are P uptake-related features [e.g., root length (RL) and root diameter (RD)] and the degree of MR differs widely among plant species and even among plant genotypes (Chu et al., 2013; Comas et al., 2014; Kramer-Walter et al., 2016). For mycorrhizal traits, mycorrhizal colonization might be a useful parameter for breeding maize varieties with high MR (Chu et al., 2013). Root external fungal structures and the internal to external hyphae ratio (IEHR) exhibited great variation among different varieties and these two indicators may also contribute to MR (Chu et al., 2013; Sawers et al., 2016). For root traits, poorly adapted varieties with limited ability to explore the soil (e.g., large diameter roots and poorly branched roots) often show a large performance increase with AM symbiosis (Hetrick et al., 1992; Newsham et al., 1995; Comas et al., 2014). Thus, breeding higher MR crop genotypes could be an important way to enhance mycorrhizal benefits. To explore the variation pattern of MR, based on greenhouse experiments of 20 wheat cultivars, a previous study firstly proposed that the release date of the cultivar could be a decisive factor (Hetrick et al., 1992) and some later studies also verified the findings of this study (Zhu et al., 2001; Chu et al., 2013). However, for other crops, such as onion, modern breeding practices did not change mycorrhizal responses (Galvan et al., 2011). A meta-analysis of studies from 1981 to 2010 found no evidence that new crop plant genotypes lost their ability to respond to mycorrhizae due to agricultural and breeding practices (Lehmann et al., 2012). Therefore, it is clear that previous mainstream study cannot draw a general conclusion about the effect of modern breeding on MR and, thus, study on specific species at different P levels is needed to better predict the MR of crop genotypes.

Cotton (*Gossypium* spp.) is an economically important crop worldwide, both in providing a significant amount of natural fiber for the manufacture of textiles and as an oilseed (Bao et al., 2019). Upland cotton (*Gossypium hirsutum* L.), which belongs to one of the four cotton species, currently accounts for more than 90% of global cotton fiber production. Xinjiang Province tops China in cotton planting areas and its cotton is famous for its long pile, good quality, and high output. In the past few decades, many cotton varieties have been cultivated in Xinjiang. AM fungi can colonize up to 90% of cotton root length and enhance cotton plant P uptake or fiber yield (Cely et al., 2016; Eskandari et al., 2016). Unlike other crops, such as maize, cotton plants have fewer root exudates and, thus, depend on root morphological characteristics to obtain nutrients (Wang et al., 2010). Therefore, a study was planned to identify cotton genotypes highly responsive to AM fungal inoculation. The primary objective of this study was to determine how MR on a set of cotton genotypes has been affected by their release date from 1950 to present (“old” vs. “modern” genotypes) at different P levels (low vs. high). The second objective was to identify the relationship between MR and functional traits that are related to P uptake efficiency. Such information will provide useful information for future cotton breeding aimed at delivering crop genotypes that are less dependent on the input of P fertilizers.

## Materials and Methods

### Study Site, Soils, and Experimental Conditions

A pot experiment was conducted in a glasshouse at the Cotton Experimental Station (44°17′57″N, 86°22′6″E, 400 m.a.s.l.) of the Xinjiang Academy of Agricultural Sciences in Manasi County, Xinjiang, Northwest China. Soil was collected from field plots at the Cotton Experimental Station. The basic soil properties were pH 6.4 (water:soil = 5:1), Olsen-P 13.6 mg kg^–1^, 0.658 g total P kg^–1^, organic matter 44.10 g kg^–1^,104.5 available K mg kg^–1^, and total N 2.35 g kg^–1^. Before the experiment, the soil was air dried, passed through a 2-mm sieve, and sterilized by γ-irradiation with 25 kGy ^60^Co.

The following mineral nutrients were mixed uniformly (kg^–1^ soil) with the soil before the start of the pot experiment: 200 mg N (as NH_4_NO_3_), 200 mg K (as K_2_SO_4_), 50 mg Mg (as MgSO_4_⋅7H_2_O), 5 mg Zn (as ZnSO_4_⋅7H_2_O), 5 mg Mn (as MnSO_4_⋅H_2_O), and 2 mg Cu (as CuSO_4_⋅5H_2_O). Each pot was 23 cm in height and 18 cm in diameter and contained 3 kg of sterilized soil.

### Arbuscular Mycorrhizal Fungal Inoculation

The indigenous AM fungal community (including spores and hyphae) was sieved from the farmland soil. To obtain sufficient AM fungal inocula, the indigenous fungal community in the soil was propagated in a 5:1 mixture (w/w) of zeolite and river sand with maize for 4 months in a greenhouse and the inocula contained spores (15 spores g^–1^ of soil), mycelium, and fine root segments. A 40-g dry weight AM fungal inoculum and sterilized inoculum were added to mycorrhizal and nonmycorrhizal pots, respectively. In addition, to correct for differences in other soil bacterial communities between the mycorrhizal and nonmycorrhizal treatments, a 10-ml soil suspension filtered from AM general inocula in deionized water was added to each nonmycorrhizal pot (Hodge et al., 2001) and 10 ml of deionized water was added to each mycorrhizal pot.

### Test Plants

A total of 24 cotton varieties were used as host plants: C-3174 (C3), KK-1543 (KK), 108 Fu (108), C-4744 (C4), Che 61-72 (CHE), Nongkeng 5 (N5), and TM-1 (TM) were bred in the 1950s and were considered the old genotypes; Tashigan 2 (T2), Xinluzao1 (Z1), Junmian 1 (J1), Xinluzao 2 (Z2), SuK 202 (SK), and Xinluzhong 4 (Z4) were bred between 1970 and 1990; Xinlu 201 (X201), Xinluzao 13 (Z13), Xinluzao 19 (Z19), Xinluzhong 21 (ZH21), Xinluzao 31 (Z31), Xinluzhong 35 (ZH35), Xinluzhong 40 (ZH40), Xinluzao 48 (Z48), Xinluzao 50 (Z50), Xinluzhong 54 (ZH54), and Xinluzao 57 (Z57) were bred after 2000 and were considered the modern genotypes (Supplementary Table 1). TM-1 is genetic standard line and the other 23 cotton varieties are hybrids.

In each pot, three cotton seeds (surface sterilized for 10 min in 10% H_2_O_2_ and germinated in the dark) were sown on May 26, 2017; they were thinned to one plant per pot after germination. During the experiment, deionized water was supplied daily to adjust the soil moisture content to 18% (w/w). The pots were placed in random blocks and rearranged every week. The plants were harvested on July 26, 2017.

### Experimental Design

The pot experiment was conducted under a complete factorial design. There were 24 cotton genotypes (Supplementary Table 1), two P levels (15 and 150 mg P kg^–1^ soil as KH_2_PO_4_, hereafter referred to as P15 and P150, respectively), and two mycorrhizal treatments (inoculation with indigenous AM fungi or not). An appropriate P level (P15) was set to meet the demand for P in cotton growth and an extremely excessive P level (P150) was set to detect the MR of cotton genotypes at different P levels. Each of the 96 treatment combinations was replicated four times to give a total of 384 pots.

### Harvest and Sample Analysis

Plants were harvested after 8.5 weeks. At harvest, shoots, roots, and soil were separately collected. Shoots were oven dried at 70°C for 3 days and weighed. Dry shoots were ground into powder with a grinder, 0.2 g of each sample was digested in H_2_SO_4_ and H_2_O_2_ solutions, and the ammonium molybdate colorimetric method was used to quantify the P content in the shoots (Thomas et al., 1967).

Roots were carefully removed from the soil, gently washed with deionized water, and stored at −20°C. A series of root-relevant indicators, such as root length and diameter, were scanned and measured using the WinRHIZO scanning and image-recording system (EPSON 1680, WinRHIZOPro2004b). The “percentage root length” (PRL) indicated the percentage of the root length with different RD to the total root length. The PRL was calculated to reflect the distribution of root length in fine roots and thick roots. From the stored root subsamples, DNA was extracted from 0.1 g fresh cotton roots with the Plant DNA Isolation Reagent following the instructions of the manufacturer (Tiangen Biotech, Beijing, China). The AM fungal gene copies in roots were detected to assess the AM fungal root colonization rate (Zhou et al., 2020). The preparation of standards was performed according to Kiers et al. (2011). Then, standard and sample DNA was amplified using the 12.5 μl Biomed q-PCR SYBR Mix (Toyobo, Japan), 1.0 μl BSA, 7.5 μl ddH_2_O, 1.0 μl forward primer [AMV4.5NF, 5′-CGCCCGCCGCGCGCGGCGGGCGGGGCG GGGGCACGGGGGG (GC clamp) AAGCTCGTAGTTGAATT TCG-3′], and 1.0 μl reverse primer (AMDGR, 5′-CCC AACTATCCCTATTAATCAT-3′) in a 25-μl reaction (Sato et al., 2005). PCR amplification was performed under the following conditions with a Q-TOWER (Jena, Germany, United Kingdom): DNA was predenatured at 94°C for 5 min, followed by 40 cycles of denaturation at 94°C for 30 s, annealing at 60°C for 45 s, and extension at 72°C for 1 min. After each cycle, the fluorescence was detected and the dissolution curve was collected. The DNA copy number of each sample was calculated according to the standard curve.

The soil remaining in each pot was fully mixed. Subsamples were air dried and used to determine the hyphal length density (HLD) (meters of hyphae per gram of soil). A 2-g soil were blended at high speed in a Waring Blender with 250 ml of deionized water for 30 s. The blended suspension was rapidly transferred to wide-necked Erlenmeyer flasks; these were agitated vigorously by hand shaking and left on the bench for 60 s. Duplicate 5 ml aliquots were pipetted onto 25 mm Millipore filters in a filtration manifold holding 10 filters. The filters were covered with lactoglycerol-trypan blue for 5 min and transferred to microscope slides to dry (Johansen et al., 1993). The HLD was calculated by recording the number of stained hyphae crossing the grid.

Internal to external hyphae ratio was used to indicate the relative length of the intra- and extraradical cellular mycelia (Chu et al., 2013). IEHR was calculated as follows:


$$\text{IEHR}=\,\frac{\mathrm{Root}\,\mathrm{length}\times M\times \mathrm{Root}\,\mathrm{diameter}}{\mathrm{Hyphal}\,\mathrm{diameter}\times \mathrm{Hyphal}\,\mathrm{length}}$$

where M refers to mycorrhizal colonization, assessed by AM fungal gene copy number.

### Mycorrhizal Responsiveness

In this study, MR included mycorrhizal growth responsiveness (MGR) and mycorrhizal P responsiveness (MPR). MR was defined according to Janos (2007):


MR=M-NC

where M is the trait value for colonized plants and NC is the trait value for noncolonized plants.

### Data Analysis

All the statistical analysis data were checked for homogeneity of variances using Levene’s tests (*p* > 0.05). The specific copy numbers of AM fungi were log-transformed before statistical analysis. The main effects of treatments and their interactions were tested using the three-way ANOVA (Supplementary Table 2). The two-way ANOVAs were carried out to test for the effects of P levels and cotton genotypes on MGR and MPR. Significant differences between the old and modern cotton genotypes in the P levels were compared by the Wilcoxon signed-rank test at *p* ≤ 0.05. Differences were statistically significant at *p* ≤ 0.05. Principal component analysis (PCA) was performed with R statistics using dudi.pca in the ade4 package (Dray and Dufour, 2007) using centered and scaled data to determine the relationships among plant traits and mycorrhizal traits.

## Results

### Mycorrhizal Colonization and the Hyphal Length Density

**FIGURE 1:**
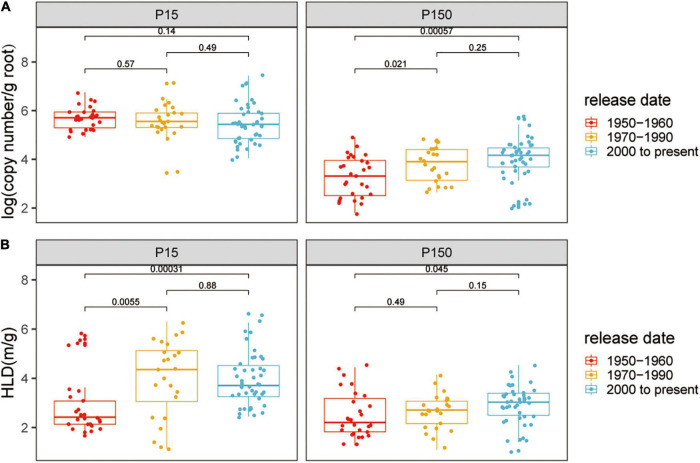
**(A)** Arbuscular mycorrhizal (AM) fungal gene copy number and **(B)** the hyphal length density (HLD) of cotton varieties inoculated with indigenous AM fungi under low or high phosphorus (P) supply. The groups (and bar patterns) are as follows: 1950–1960 (red), 1970–1990 (yellow), and 2000 to present (blue). We define those from “1950–1960” and “2000 to present” as the old and modern varieties, respectively. Boxes show first quartile, median, and third quartile. Whiskers extend to the most extreme points within 1.5 × box lengths and the points are values that fall outside the whiskers. The Wilcoxon signed-rank test was carried out among different groups and the *p*-value is marked.

The AM fungal gene copy number in roots was detected to indicate the colonization of AM fungi. Quantitative real-time PCR analysis indicated that cotton plants inoculated with indigenous AM fungi were successfully colonized, while plants without inoculation were free of mycorrhizal association (CT values were not detected). Compared with the low P condition, the copy number of colonization with AM fungi was lower (low P, mean = 5.56; high P, mean = 3.74; *p* < 0.05) (Supplementary Figure 1) and showed higher variation (low P, CV = 0.13, high P, CV = 0.23; *p* < 0.05) at high P. However, the ability of the plants to form AM symbiosis still existed despite the high P supply. At both the P levels, AM fungal gene copy number significantly differed among the genotypes, reflecting the genetic diversity of cotton plants in response to mycorrhizal fungi (Supplementary Figure 1A). To further distinguish the impact of the release date of mycorrhizal characteristics, 24 varieties were divided into three groups according to the date of breeding: 1950–1960 (defined as the old genotypes), 1970–1990, and 2000 to present (defined as the modern genotypes, Supplementary Table 1). Based on release date, the root AM fungal gene copy number tended to gradually increase at high P, whereas at low P, the root AM fungal gene copy number did not show distinct variation with the release date of the cotton varieties (Figure 1A).

The HLD reflected the successful extraradical mycelial growth of AM fungi outside the host root. The HLD of cotton without AM fungal inoculation was negligibly low. The growth of the HLD significantly decreased with the supply of P (low P, mean = 4.05 m g^–1^; high P, mean = 2.85 m g^–1^; *p* < 0.05) (Supplementary Figure 1B). The HLD significantly differs among the genotypes within each P supply and the HLD of ZH35 and C3 was always relatively higher at low or high P levels (Supplementary Figure 1B). Notably, at both the low and high P, the modern genotypes had the higher HLD than the old genotypes, corresponding to the results of AM fungal gene copy number (Figure 1B).

### Root Architecture Characteristics

**FIGURE 2:**
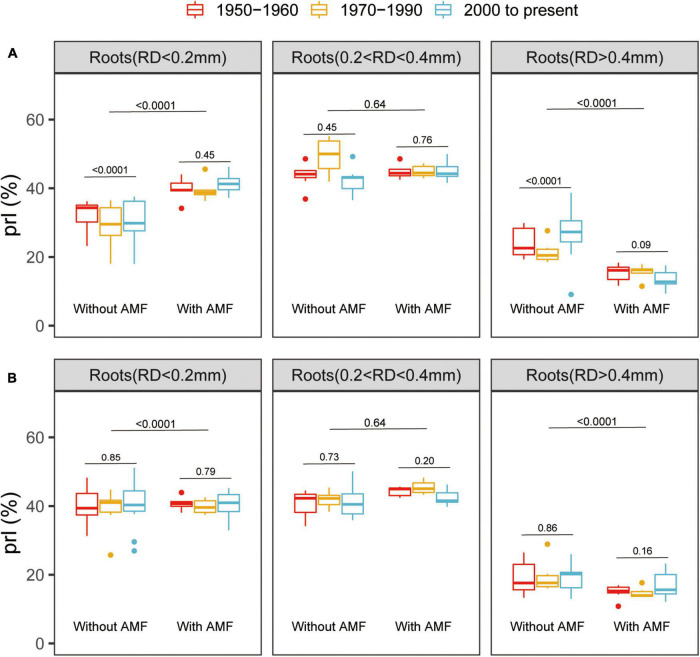
Box plots showing the percentage root lengths (PRLs) with different root diameters (RD) for the total root length of cotton genotypes grown under low **(A)** or high **(B)** P supply and with or without inoculation with indigenous AM fungi. The groups (and bar patterns) are as follows: 1950–1960 (red), 1970–1990 (yellow), and 2000 to present (blue). We define those from “1950–1960” and “2000 to present” as the old and modern varieties, respectively. Boxes show first quartile, median, and third quartile. Whiskers extend to the most extreme points within 1.5 × box length. The Wilcoxon signed-rank test was carried out between the old and modern varieties or between with and without inoculation with indigenous AM fungi and the *p*-value is marked.

All the root traits were significantly affected by variety, AM fungal inoculation, and P supply level (Supplementary Table 2). Mycorrhizal inoculation had a significant positive effect on root development, remarkably enhancing RL, specific root length (RD < 0.2 mm), and root surface area (RS) for all the genotypes (Supplementary Figure 2). Furthermore, at low P, the modern varieties without AM fungal inoculation exhibited greater RL, specific root length (RD < 0.2 mm), and RS than the old varieties (Supplementary Figure 2). At high P, when inoculated with AM fungi, the old varieties exhibited greater RL, specific root length (RD < 0.2 mm), and root surface area than the modern varieties (Supplementary Figure 2). Mycorrhizal plants had significantly more thin roots (RD < 0.2 mm) and significantly fewer thicker roots (RD > 0.4 mm) at both the low and high P (Figure 2). Interestingly, there was no significant difference in the PRL between the old and modern cotton genotypes when inoculated with AM fungi, suggesting that AM-mediated dynamics in the root traits remained the same despite the release date (Figure 2).

### Modern Cotton Genotypes Benefited More From Plant-Arbuscular Mycorrhizal Fungi Association

A total of 24 cotton genotypes released in different dates, from the 1950s to the 2010s, were evaluated for MR with the inoculation of indigenous AM fungi (M) or without inoculation (NC) (Supplementary Table 1). Both the shoot dry weight (SDW) and shoot P (SP) content were significantly affected by genotypes, mycorrhizae, and P levels, but their interactions were not significant for SDW (Supplementary Table 2). For all the cotton genotypes, SDW increased with greater P addition without AM fungi, indicating that the plants were in P demand condition at low P supply (Supplementary Table 3). In the presence of mycorrhizae, all the varieties exhibited enhancement in SDW and SP, irrespective of P supply, suggesting that mycorrhizae were beneficial for plant growth (Supplementary Table 3).

**FIGURE 3:**
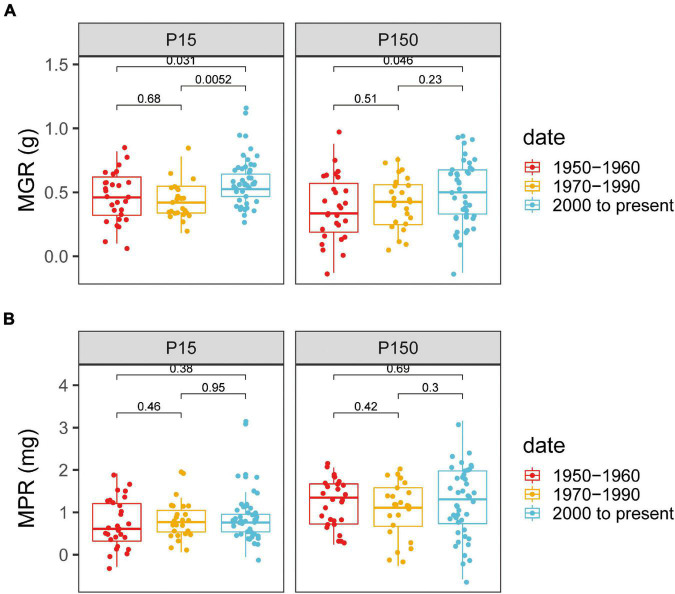
Mycorrhizae-induced changes in shoot growth [mycorrhizal growth responsiveness (MGR)] and shoot P content [mycorrhizal P responsiveness (MPR)] for cotton varieties with different release dates under low **(A)** or high **(B)** P supply. The groups (and bar patterns) are as follows: 1950–1960 (red), 1970–1990 (yellow), and 2000 to present (blue). We defined those from “1950–1960” and “2000 to present” as the old and modern varieties, respectively. Boxes show the first quartile, median, and third quartile. Whiskers extend to the most extreme points within 1.5 × box length. The Wilcoxon signed-rank test was carried out between different groups and the *p*-value is marked.

Mycorrhizal growth responsiveness and MPR were significantly affected by P supply levels and varieties and by the interaction of genotypes × P levels (Supplementary Table 2). There was an obvious distinction between the old and modern genotypes for MGR (Figure 3A). At both the P levels, all the chosen cotton varieties inoculated with AM fungi showed a positive MGR (Supplementary Table 3). At low P levels, the modern varieties, with a panel-wide MGR of 0.57 ± 0.06 g (mean ± SE), had significantly greater MGR than the old varieties, with a panel-wide MGR of 0.42 ± 0.07 g (Figure 3A). Similar scenarios appeared under high P supply, in which the MGR_modern varieties_ was 0.49 ± 0.04 g, which was still significantly higher than the MGR_old varieties_ (0.38 ± 0.04 g) (*p* < 0.05, Figure 3A).

Mycorrhizae-induced changes in shoot P content (MPR) had high variation within the released group dates, but these genotypes demonstrated no obvious specific pattern (Figure 3B). Plants inoculated with AM fungi had a high plant growth response, but with no observed increase in shoot P content, whereas the P concentration in the mycorrhizal plants was reduced compared with that in nonmycorrhizal plants (Supplementary Table 4). The reduced P concentration may be an indication of a dilution effect of P in mycorrhizal plants.

### Mycorrhizal Responsiveness Was Positively Correlated With the Hyphal Length Density at Low P

There were substantial variations in MR among genotypes belonging to the modern or old groups for MGR and MPR under both the low and high P supply. Considering the P status in arable land in China at present (Li et al., 2011), the modern genotypes that showed better MR at low P levels (Olsen-P was 25 mg/kg) were paid more attention to identify the genotypes with the highest MR, thus far released that demonstrated a strong relation with AM fungi and facilitated plant growth. The top five genotypes by MGR included C3, ZH35, Z19, Z31, and Z57, which are in bold (Supplementary Table 3). Notably, among them, four are modern genotypes, which clearly demonstrate that the modern genotypes are more mycorrhizal dependent on plant growth than the old genotypes.

**FIGURE 4:**
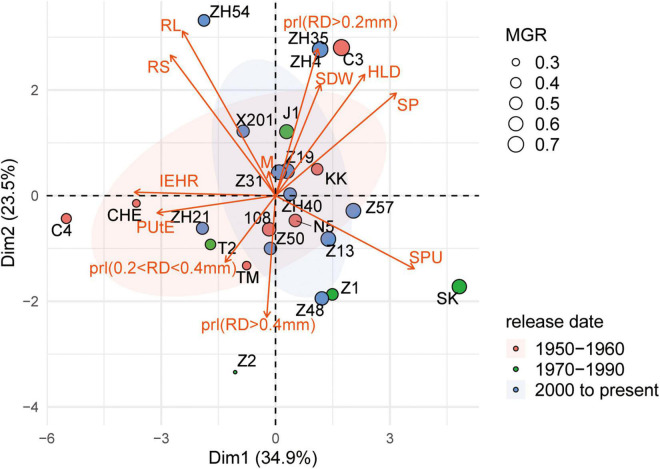
Principal component analysis of plant growth, morphological traits, and mycorrhizal traits of 24 cotton varieties grown under low P supply and inoculated with indigenous AM fungi. Biplot showing scores in the first two principal components (PC1, *x*-axis; PC2, *y*-axis) for traits [red arrows: shoot dry weight (SDW), shoot P concentration (P conc.), mycorrhizal colonization indicated by AM fungal gene copy number (M), HLD, root length of different diameters, root diameter (RD), root length (RL), root volume (RV), shoot P (SP) content, P utilization efficiency (PUtE), and specific P uptake (SPU) in shoots by unit of root length]. In addition, we calculated the PRL, the percentage of the root length with different root diameters. Points are colored by release date. The size of point indicates the mycorrhizal growth response (MGR, g), calculated as the difference in SDW in colonized and noncolonized plants.

**FIGURE 5:**
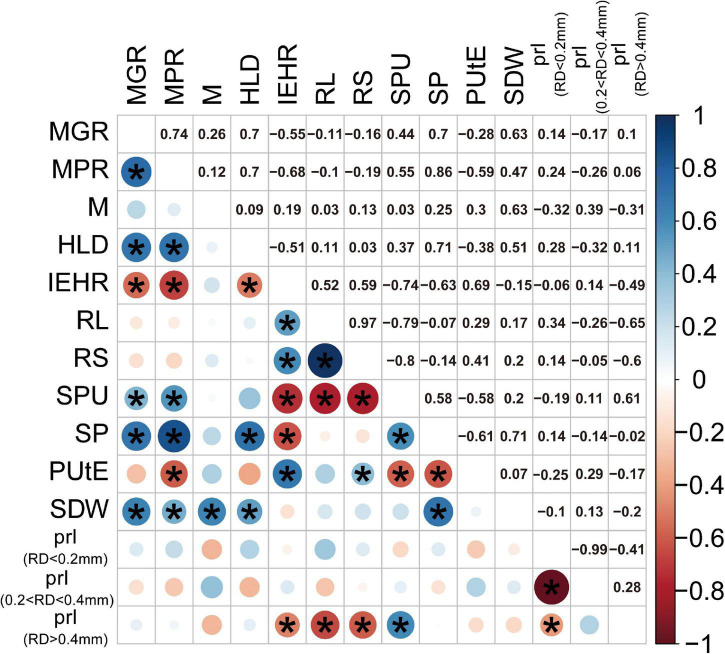
Correlation matrix (Pearson) of SDW (g), SP content (mg), mycorrhizal responsiveness (MGR and MPR), root morphological traits, and mycorrhizal traits at low P levels. Blue circles indicate a positive correlation, red circles indicate a negative correlation, and the size and intensity of shading indicate magnitude. Correlations significant at *p* < 0.05 are marked with an asterisk. Corresponding measurements of each abbreviation: mycorrhizal colonization indicated by AM fungal gene copy number (M), HLD, RS, total RL, PUtE, SPU in shoots by unit of root length, mycorrhizal responsiveness (MGR and MPR), SDW, and SP content. In addition, we calculated the PRL, the percentage of the root length with different diameters.

To examine different attributes of cotton genotypes in response to low P and AM fungi, root morphology and mycorrhizal traits among 24 cotton genotypes were further analyzed. A PCA was performed using data specifically for the mycorrhizal plants at low P by integrating these data with plant growth and mycorrhizal response (Figure 4). In addition, the pairwise correlations among all the measurements were calculated (Figure 5). The first two principal components (PCs) captured 60% of the trait variation (PC1: 35%; PC2: 24%) and separated the 24 genotypes based on the magnitude of MGR rather than release date (Figure 4 and Supplementary Table 5). The SDW was associated in the PC space with AM fungal gene copy number, the HLD, and shoot P content (Figure 4, right upper quadrant and Figure 5), but not related to the PRL (Figures 4, 5). To characterize P absorption efficiency, we plotted “specific P uptake” (SPU), which reflects P uptake in shoots by unit of root length. SPU was positively related to shoot P content (Figure 5). In this study, the ratio of plant P content to shoot biomass was used to indicate P utilization efficiency (PUtE). When the plants were mycorrhizal, PUtE was decreased and remarked negatively related to shoot P content (Supplementary Figure 3 and Figure 5). Placing the genotypes on the PC space, genotypes with high MR (bigger dots), including most modern genotypes, were separated from those with low MR (smaller dots) on the basis of PC2 (Figure 4). The second principal component (PC2) was mainly explained by the RL, RS, PRL (RD < 0.2 mm), and the HLD (Supplementary Table 5). Combined with the results of direct calculation of pairwise relationships (Figure 5), RL and RS had a greater contribution to PC2, but no significant correlation with MR (Supplementary Figure 2). Overall, MR was positively associated with the HLD at low P (Figure 4). These results explained the higher MR of the modern varieties described above: the modern varieties were highly responsive to inoculation with indigenous AM fungi compared to the old varieties with the higher HLD in the inoculated plants.

## Discussion

Extensive studies have revealed that MR and its trends based on release dates vary greatly among crop genotypes. For instance, the MR of old wheat cultivars was higher than that of modern wheat cultivars (Hetrick et al., 1992, 1996; Zhu et al., 2001). Nevertheless, MR for maize, tomato, and oats showed the opposite scenario (Koide et al., 1988; Bryla and Koide, 1998). Generally, the release date of a cultivar reflects the agricultural and breeding practices of its time (Lehmann et al., 2012). In the past few decades in Xinjiang, cotton breeding has mainly aimed to increase quality, fiber yield, and disease resistance, but has not considered how modern varieties interact with indigenous AM fungi. The data presented in this study showed that mycorrhizal colonization and the HLD of the modern cotton genotypes were relatively higher than those of the old genotypes at low and high P (Figure 1 and Supplementary Figure 1). The mycorrhizal colonization and the HLD of cotton genotypes developed between the 1980s and the 1990s exhibited an intermediate status between the old and modern genotypes (Figure 1 and Supplementary Figure 1). The 24 highly diverse cotton genotypes analyzed showed a positive response to AM fungi, irrespective of the initial soil P levels, indicating the ability of cotton to form a beneficial symbiosis with AM fungi (Figure 2 and Supplementary Table 3). The 24 cotton genotypes were divided into three groups according to release date, we noticed that MGR showed an increasing trend (Figure 3). Similar to maize, tomato, and oats (Koide et al., 1988; Bryla and Koide, 1998), the modern cotton genotypes benefitted more from plant growth in association with AM fungi than the old genotypes at both the P levels (Figure 3). Overall, the results indicated that release date had significant positive effects on the formation of AM symbiosis and on the MGR of modern cotton genotypes.

Enhancement of P uptake efficiency (PUpE) and PUtE is widely suggested as a target of crop breeding (Hetrick et al., 1992; Zabinski et al., 2002; Lynch, 2011; Leiser et al., 2016; Ros et al., 2018; Wang et al., 2020a). Increase in shoot P concentrations and decrease in PUtE (defined as the inverse of P concentration) for wheat, maize, and *Centaurea* after AM fungal inoculation were common phenomena in previous study (Hetrick et al., 1992; Zabinski et al., 2002; Wang et al., 2020a). This phenomenon has been described as luxury uptake in previous studies, but the term luxury uptake has no explanatory value (Hetrick et al., 1994; Zabinski et al., 2002). A further explanation is that P uptake is not perfectly regulated over time and “excess” P can be used at later stages (Wang et al., 2020a). Compared with previous results above, the results in this study showed an opposite trend: decrease in shoot P concentrations and increase in PUtE in most cotton varieties after AM fungi inoculation (Supplementary Figure 3 and Supplementary Table 4). Mycorrhizal plants had higher biomass than nonmycorrhizal plants (Supplementary Table 3). AM fungi may provide other nutrients to plants and, thus, P is more dilute in the tissues. The reason for the increase in PUtE after inoculation remains to be explored.

Plant genotypes with different root structures and functions are crucial for understanding the large variation in MR from positive to negative (Hetrick, 1991; Hao et al., 2008; Yucel et al., 2009). Traditionally, plant varieties with a confined and coarse root architecture, such as relatively low root hair density, large diameter roots, and short root hairs, obtain the greatest growth benefits from colonization by AM fungi (Brundrett, 2002; Ah, 2004; Smith and Read, 2008). Nonetheless, a meta-analysis combined previously published literatures indicated that the relationships between MGR and allocation to roots, RD, root hair length, and root hair density was statistically insignificant (Maherali, 2014). Similarly, a recent study indicated that magnitudes and directions of MR of 27 crops were diverse among crop species and were unrelated to the coordinated evolution with fine root traits (Martin-Robles et al., 2018). The particularity of the cotton root system is that it is mainly composed of fine roots: the abundance of fine roots (RD < 0.4 mm) occupied more than 70% (Figure 2). But, for other crops, such as maize, the proportion of fine root length was 10–20%, which was significantly lower than that of cotton (Wang et al., 2020a). When colonized with AM fungi, cotton plants had a notably higher abundance of roots with RD < 0.2 mm, but a decreased abundance of roots with RD > 0.4 mm, which was clear evidence that these root traits are functions of AM fungi (Figure 2). Plants need to invest more photosynthetically-fixed carbon to grow thick roots than thin roots (Kaschuk et al., 2009). When inoculated with AM fungi, the plants could then allocate more photosynthetically-fixed carbon to AM fungi and less photosynthetically-fixed carbon to roots (Smith and Read, 2008; Kaschuk et al., 2009). The reduction in carbon allocation to the root has led to the increased abundance of fine roots and the decreased abundance of thick roots in this study (Figure 2). Moreover, the MPR had a significant positive correlation with the abundance of root lengths with RD < 0.2 mm. Altogether, cotton plants in association with AM fungi had a unique root morphological trait and a greater abundance of fine roots was a predictor of MPR. However, when combined with the nonsignificant relationship between fine roots and MGR (Figure 5), further experiments are needed to verify the stability of the relationship between the PRL and MR.

It has been suggested that the variation of plant genetic factors may influence the degree of AM fungal colonization and, thus, determine the variation in growth response to AM fungi. Much emphasis has been invested in study on the development of root-internal fungal structures, as more arbuscules may transfer more mineral nutrients, such as P, to the host plant (Lehmann et al., 2012; Chu et al., 2013). The length of root-external hyphae may reflect the ability to explore mineral nutrients from the root free soil. The recent study showed that the apparent benefits of AM fungi for plant growth and P uptake were correlated with the increased abundance of root-external hyphae rather than root colonization (Sawers et al., 2016). It was tempting to speculate that the extension of mycelium, coupled with the ability of AM fungi to absorb mineral nutrients, determined the response to AM fungi (Jakobsen et al., 2001; Sawers et al., 2016). Considering that the relationship between MR and mycorrhizal colonization was nonsignificant, quantification of mycorrhizal colonization was not predictive of MR (Figure 5). Among all the 24 cotton varieties, the HLD was positively correlated with MGR and MPR in this study (Figure 5). The higher HLD of the modern cotton genotypes contributed to the increase in MR compared to the old genotypes. It is supposed that the variation of genetic factors between old and modern cotton genotypes may have a great impact on the supply of carbon to the AM fungi, which, in turn, affected the growth of root-external hyphae and even the magnitude of MR (Keymer and Gutjahr, 2018; Berger and Gutjahr, 2021). Overall, it could, thus, be suggested that the HLD is necessarily a good predictor of cotton growth response to AM fungal colonization.

## Conclusion

The results of this study show that modern cotton genotypes are more intensely colonized and more mycorrhizae responsive than the old genotypes. It can be concluded that modern breeding in Xinjiang Province has improved the association of cotton varieties with indigenous AM fungi. Thus, harnessing indigenous AM fungi as beneficial microbes presents a promising strategy to strengthen the functionality of AM symbiosis, thereby reducing the dependence of crop production on adding P chemical fertilizers and optimizing agricultural sustainability. Moreover, the HLD can be used as a proxy for the MR of cotton genotypes based on its correlation with MGR. These results provide useful information for future plant breeding programs aimed at developing crops that benefit from AM fungi.

## Data Availability Statement

The original contributions presented in the study are included in the article/Supplementary Material, further inquiries can be directed to the corresponding author.

## Author Contributions

GF, LW, and XW designed the experiment. LW, XW, and BM conducted experiments and completed the measurement of the experimental indicators. LW conducted all analyses, conceived figures, and wrote the manuscript. GF, LW, XW, AK, and KK contributed to revisions. All authors contributed to the article and approved the submitted version of the manuscript.

## Conflict of Interest

The authors declare that the research was conducted in the absence of any commercial or financial relationships that could be construed as a potential conflict of interest.

## Publisher’s Note

All claims expressed in this article are solely those of the authors and do not necessarily represent those of their affiliated organizations, or those of the publisher, the editors and the reviewers. Any product that may be evaluated in this article, or claim that may be made by its manufacturer, is not guaranteed or endorsed by the publisher.
