# Supplementary material for: Breeding Practice Improves the Mycorrhizal Responsiveness of Cotton (Gossypium spp. L.)
Source: Front Plant Sci. 2021 Dec 10;12:780454. doi: 10.3389/fpls.2021.780454 (PMC8703140; doi:10.3389/fpls.2021.780454)
Supplement: Supplementary file 1 [file Data_Sheet_1.docx]

Supplementary Material

| **Genotypes** | **Logogram** | **Year of release** | **Hybrid parent** | **Group** |
| --- | --- | --- | --- | --- |
| C-3174 | C3 | 1950 |  | 1950-1960  **(old genotypes)** |
| KK-1543 | KK | 1955 | СЧ2 × KK-351 |  |
| 108 Fu | 108 | 1955 |  |  |
| C-4744 | C4 | 1956 |  |  |
| Che 61-72 | CHE | 1961 |  |  |
| Nongkeng 5 | N5 | 1969 | Liao4086 × Liao1038 |  |
| TM-1 | TM | 1970 | genetic standard line |  |
| Tashigan 2 | T2 | 1978 |  | 1970-1990 |
| Xinluzao1 | Z1 | 1978 | 20-8 × SM |  |
| Junmian 1 | J1 | 1979 | C1470 × Wuyidaling |  |
| Xinluzao 2 | Z2 | 1988 | 6902 × Zhongmiansuo 4 |  |
| SuK 202 | SK | 1991 |  |  |
| Xinluzhong 4 | ZH4 | 1992 | Daizi 45 × Xinlu 202 |  |
| Xinlu 201 | X201 | 2002 |  | 2000 to present  **(modern genotypes)** |
| Xinluzao 13 | Z13 | 2002 | 83-14 × 5601 and 1693 |  |
| Xinluzao 19 | Z19 | 2004 | 91-2 × 900 |  |
| Xinluzhong 21 | ZH21 | 2004 | 95 × 96-07 |  |
| Xinluzao 31 | Z31 | 2007 | Aizimian × Beiersinuo |  |
| Xinluzhong 35 | ZH35 | 2008 | XY36 × Jimian 123 |  |
| Xinluzhong 40 | ZH40 | 2009 | 96 Fu 56 × Xinkai 99-10 |  |
| Xinluzao 48 | Z48 | 2010 | Shixuan 87 × Youxi 604 |  |
| Xinluzao 50 | Z50 | 2011 | 97-55 × 225 |  |
| Xinluzhong 54 | ZH54 | 2012 | K-265 × K-263 |  |
| Xinluzao 57 | Z57 | 2013 | 60-2 × Xinluzao 8 |  |

**Supplementary Table 1** List of 24 cotton genotypes bred from 1950 to the present and divided into 3 groups according to release date. We define those from “1950-1960” and “2000 to present” as the old and modern genotypes, respectively.

| **Traits** | **Genotypes (V)** | **Inoculation (I)** | **Phosphorus (P)** | **V*I** | **V*P** | **I*P** | **V*I*P** |
| --- | --- | --- | --- | --- | --- | --- | --- |
| Shoot dry weight (SDW) | 3.501** | 534.152** | 92.336** | 1.527 | 1.345 | 2.738 | 0.893 |
| Shoot P concentration (P conc.) | 4.511** | 49.166** | 302.902** | 2.136** | 1.154 | 12.819** | 1.819** |
| P utilization efficiency (PUtE) | 4.264** | 52.406** | 290.804** | 1.479 | 1.543 | 30.549** | 1.905** |
| Shoot P content (SP) | 1.499** | 407.692** | 293.770** | 1.748* | 2.211 | 11.518** | 1.082 |
| Specific P uptake (SPU) | 3.814** | 4.662* | 136.769** | 1.593* | 3.114** | 65.256** | 1.336 |
| Root length (RL) | 3.883** | 278.221** | 32.082** | 2.313** | 1.683* | 144.991** | 0.774 |
| Root surface area (RS) | 3.955** | 226.143** | 25.642** | 2.335** | 1.404 | 107.581** | 0.748 |
| Mycorrhizal colonization (M) | 11.420** |  | 832.285** |  |  | 9.363** |  |
| Hyphae length density (HLD) | 5.794** |  | 63.076 ** |  |  | 5.995** |  |
| Mycorrhizal growth response (MGR) | 4.742** |  | 8.512** |  |  | 2.773** |  |
| Mycprrhizal P uptake response (MPR) | 5.402** |  | 35.595** |  |  | 3.345** |  |

**Supplementary Table 2** ANOVA results for the effects of genotypes, AM fungal inoculation, P levels, and their interactions on cotton growth, root and mycorrhizal traits. F values are shown. **P*<0.05; ***P*<0.01; indicating significance.

| Varieties | P15 | | | | | |  | P150 | | | | | |
| --- | --- | --- | --- | --- | --- | --- | --- | --- | --- | --- | --- | --- | --- |
|  | Shoot dry weight (g) | | | Shoot P content (mg) | | |  | Shoot dry weight (g) | | | Shoot P content (mg) | | |
|  | NC | M | MGR(SE) | NC | M | MPR(SE) |  | NC | M | MGR(SE) | NC | M | MPR(SE) |
| **C3** | 0.53 | 1.27 | 0.74 (0.04)ab | 1.16 | 2.61 | 1.46 (0.17)a..f |  | 0.69 | 1.22 | 0.53 (0.09)a..g | 1.96 | 3.55 | 1.59 (0.2)a..e |
| KK | 0.64 | 1.09 | 0.45 (0.12)a..g | 1.39 | 2.27 | 0.88 (0.25)a..g |  | 0.85 | 1.04 | 0.19 (0.15)d..g | 2.19 | 2.85 | 0.66 (0.26)b..g |
| 108 | 0.55 | 1.11 | 0.56 (0.03)a..g | 1.28 | 1.93 | 0.64 (0.13)b.g. |  | 0.94 | 1.12 | 0.18 (0.03)fg | 2.22 | 2.87 | 0.65 (0.12)b..g |
| C4 | 0.69 | 1.07 | 0.38 (0.06)a..g | 1.42 | 1.56 | 0.14 (0.16)fg |  | 1.11 | 1.34 | 0.22 (0.05)d..g | 2.35 | 4.26 | 1.91 (0.08)ab |
| CHE | 0.59 | 0.9 | 0.31 (0.07)a..g | 1.17 | 1.55 | 0.39 (0.12)d..g |  | 0.64 | 1.25 | 0.61 (0.09)a..g | 1.59 | 3.25 | 1.66 (0.02)a..d |
| N5 | 0.43 | 0.93 | 0.50 (0.07)a..g | 1.45 | 1.76 | 1.27 (0.21)a..g |  | 0.56 | 1.11 | 0.55 (0.1)a..g | 2.18 | 3.1 | 1.19 (0.27)a..g |
| TM | 0.58 | 0.91 | 0.33 (0.07)a..g | 0.88 | 2.15 | 0.31 (0.1)efg |  | 0.73 | 1.08 | 0.36 (0.05)a..g | 1.86 | 3.05 | 0.92 (0.16)a..g |
| T2 | 0.53 | 0.98 | 0.41 (0.03)a..g | 1.19 | 1.7 | 0.51 (0.03)c..g |  | 0.91 | 1.25 | 0.47 (0.03)a..g | 2.32 | 3.96 | 1.64 (0.19)a..d |
| Z1 | 0.58 | 0.99 | 0.46 (0.04)a..g | 1.36 | 2.02 | 0.65 (0.04)b..g |  | 0.74 | 1.37 | 0.51 (0.04)a..g | 2.46 | 3.59 | 1.13 (0.09)a..g |
| J1 | 0.57 | 1.16 | 0.59 (0.02)a..g | 1.19 | 2.23 | 1.04 (0.1)a..g |  | 0.72 | 1.33 | 0.61 (0.08)a..g | 2.03 | 3.55 | 1.52 (0.22)a..e |
| Z2 | 0.61 | 0.86 | 0.25 (0.03)c..g | 1.36 | 1.71 | 0.35 (0.14)d..g |  | 0.84 | 1.02 | 0.18 (0.02)efg | 2.66 | 2.8 | 0.14 (0.07)fg |
| SK | 0.4 | 1.01 | 0.61 (0.1)a..g | 1.09 | 2.42 | 1.33 (0.18)a..g |  | 0.6 | 0.94 | 0.34 (0.05)a..g | 1.87 | 2.93 | 1.06 (0.1)a..g |
| ZH4 | 0.67 | 1.03 | 0.36 (0.03)a..g | 1.42 | 2.32 | 0.9 (0.08)a..g |  | 0.85 | 1.26 | 0.41 (0.15)a..g | 2.43 | 3.3 | 0.87 (0.48)a..g |
| X201 | 0.47 | 0.96 | 0.49 (0.09)a..g | 1.23 | 1.93 | 0.7 (0.09)b..g |  | 0.72 | 1.16 | 0.44 (0.04)a..g | 2.34 | 2.76 | 0.42 (0.37)d..g |
| Z13 | 0.51 | 1.14 | 0.64 (0.05)a..f | 0.96 | 2.23 | 1.12 (0.13)a..g |  | 1.04 | 1.32 | 0.29 (0.07)b..g | 2.85 | 3.85 | 0.88 (0.2)a..g |
| **Z19** | 0.59 | 1.24 | 0.65 (0.02)a..d | 1.66 | 2.26 | 0.6 (0.19)b..g |  | 0.72 | 1.19 | 0.47 (0.05)a..g | 2.23 | 3.05 | 0.82 (0.08)b..g |
| ZH21 | 0.33 | 0.8 | 0.48 (0.01)a..g | 1.1 | 1.54 | 0.44 (0.06)c..g |  | 0.77 | 1.16 | 0.39 (0.13)a..g | 2.76 | 3.72 | 0.97 (0.24)a..g |
| **Z31** | 0.36 | 1 | 0.64 (0.10)a..e | 1.36 | 2.25 | 0.88 (0.14)a..g |  | 0.73 | 1.2 | 0.48 (0.09)a..g | 2.13 | 3.46 | 1.33 (0.18)a..g |
| **ZH35** | 0.4 | 1.09 | 0.7 (0.16)abc | 0.98 | 2.28 | 1.3 (0.51)a..g |  | 0.39 | 1.15 | 0.77 (0.07)a | 1.17 | 3.07 | 1.9 (0.11)ab |
| ZH40 | 0.42 | 0.93 | 0.51 (0.06)a..g | 1.04 | 1.85 | 0.81 (0.03)b..g |  | 0.46 | 1.05 | 0.59 (0.16)a..g | 2.06 | 3.63 | 1.57 (0.36)a..e |
| Z48 | 0.34 | 0.9 | 0.56 (0.08)a..g | 1.02 | 1.85 | 0.83 (0.22)b..g |  | 0.6 | 1.32 | 0.72 (0.06)ab | 2.05 | 3.93 | 1.88 (0.13)ab |
| Z50 | 0.46 | 0.96 | 0.50 (0.01)a..g | 1.22 | 1.76 | 0.55 (0.03)c..g |  | 0.79 | 0.96 | 0.17 (0.03)g | 2.68 | 2.73 | 0.05 (0.13)g |
| ZH54 | 0.58 | 1.03 | 0.46 (0.03)a..g | 1.05 | 1.98 | 0.93 (0.08)a..g |  | 0.79 | 1.52 | 0.73 (0.05)ab | 2.46 | 4.61 | 2.15 (0.09)a |
| **Z57** | 0.42 | 1.06 | 0.64 (0.07)a..f | 1.03 | 2.19 | 1.16 (0.35)a..g |  | 0.61 | 1 | 0.39 (0.18)ag | 1.66 | 3.41 | 1.75 (0.66)abc |
| average | 0.51 | 1.02 | 0.51 | 1.21 | 2.01 | 0.80 |  | 0.74 | 1.18 | 0.44 | 2.19 | 3.39 | 1.20 |

**Supplementary Table 3** Characterization of twenty-four cotton varieties grown with (NC) or without (M) inoculation with indigenous AM fungi at low or high P supply. MGR and MPR marked with different letters are significantly different among genotypes (*P* < 0.05) within a given level of P supply.

| **Traits** | **Genotypes (V)** | **Inoculation (I)** | **Phosphorus (P)** | **V*I** | **V*P** | **I*P** | **V*I*P** |
| --- | --- | --- | --- | --- | --- | --- | --- |
| Shoot dry weight (SDW) | 3.501** | 534.152** | 92.336** | 1.527 | 1.345 | 2.738 | 0.893 |
| Shoot P concentration (P conc.) | 4.511** | 49.166** | 302.902** | 2.136** | 1.154 | 12.819** | 1.819** |
| P utilization efficiency (PUtE) | 4.264** | 52.406** | 290.804** | 1.479 | 1.543 | 30.549** | 1.905** |
| Shoot P content (SP) | 1.499** | 407.692** | 293.770** | 1.748* | 2.211 | 11.518** | 1.082 |
| Specific P uptake (SPU) | 3.814** | 4.662* | 136.769** | 1.593* | 3.114** | 65.256** | 1.336 |
| Root length (RL) | 3.883** | 278.221** | 32.082** | 2.313** | 1.683* | 144.991** | 0.774 |
| Root surface area (RS) | 3.955** | 226.143** | 25.642** | 2.335** | 1.404 | 107.581** | 0.748 |
| Mycorrhizal colonization (M) | 11.420** |  | 832.285** |  |  | 9.363** |  |
| Hyphae length density (HLD) | 5.794** |  | 63.076 ** |  |  | 5.995** |  |
| Mycorrhizal growth response (MGR) | 4.742** |  | 8.512** |  |  | 2.773** |  |
| Mycprrhizal P uptake response (MPR) | 5.402** |  | 35.595** |  |  | 3.345** |  |

**Supplementary Table 4** Phosphorus concentration (mg^-1^ kg) in the shoots of twenty-four cotton genotypes grown with or without inoculation with indigenous AM fungi*.* Mean groups (lowercase letters) based on the least significant difference (Duncan) are calculated separately for NC and M plants at α = 0.05. Genotypes are ordered by release date. For every genotype, Student’s *t* test was carried out between the NC and M treatments, and the asterisk represents a significant difference (*P* < 0.05). Mean groups are calculated for NC and M plants together. Numbers in parentheses are standard errors (SE). NC: without AM fungi; M: with AM fungi.

| **Traits** | **M Low P** | | | | | | | |
| --- | --- | --- | --- | --- | --- | --- | --- | --- |
|  | **PC1 (35%)** | **PC2 (24%)** | **PC3 (19%)** | **PC4 (10%)** | **PC5 (5%)** | **PC6 (4%)** | **PC7 (2%)** | **PC8 (2%)** |
| M | -0.02 | 0.06 | -0.57 | -0.01 | -0.42 | -0.36 | -0.56 | -0.16 |
| HLD | 0.28 | 0.33 | -0.11 | 0.29 | 0.18 | 0.57 | -0.49 | 0.29 |
| IEHR | -0.44 | 0.01 | -0.08 | 0.02 | -0.15 | -0.20 | 0.07 | 0.83 |
| RL | -0.29 | 0.45 | 0.02 | 0.06 | 0.27 | -0.09 | 0.00 | -0.04 |
| RS | -0.32 | 0.39 | -0.09 | 0.04 | 0.37 | -0.08 | -0.01 | -0.08 |
| SPU | 0.43 | -0.20 | -0.10 | 0.07 | -0.04 | 0.03 | 0.12 | 0.38 |
| SP | 0.38 | 0.28 | -0.21 | 0.11 | 0.10 | -0.26 | 0.29 | 0.03 |
| PUtE | -0.37 | -0.05 | -0.23 | -0.14 | -0.33 | 0.65 | 0.17 | -0.12 |
| SDW | 0.14 | 0.30 | -0.50 | -0.01 | -0.14 | 0.10 | 0.54 | -0.04 |
| prl (RD<0.2mm) | 0.13 | 0.41 | 0.35 | -0.30 | -0.37 | -0.01 | -0.03 | 0.06 |
| prl (0.2<RD<0.4mm) | -0.15 | -0.18 | -0.02 | 0.82 | -0.09 | -0.03 | 0.11 | -0.14 |
| prl (RD>0.4mm) | -0.03 | -0.34 | -0.41 | -0.33 | 0.52 | 0.04 | -0.06 | 0.05 |

**Supplementary Table 5** Principal component analysis of plant growth, morphological traits and mycorrhizal traits of twenty-four cotton genotypes grown under low P supply and inoculated with indigenous AM fungi*.* Figures in brackets indicate the percentage of variation explained by principal components. Values indicate component loadings, which can be used to explain the meaning of the principal components. Abbreviation: mycorrhizal colonization indicated by AM fungal gene copy number (M), shoot dry weight (SDW), shoot P concentration (P conc.), percentage of root length colonized by AM fungi (M), hyphal length density (HLD), root length (RL), root surface area (RS), shoot P content (SP), P utilization efficiency (PUtE), the P uptake in shoots by unit of root length (SPU), percentage root length (prl).

**Supplementary Figure 1** Mycorrhizal colonization (A) and hyphal length density (B) of cotton genotypes under the low or high P supply inoculated with indigenous AM fungi; the cotton genotypes are listed by release date. Values are means (+SE) of four replicates. Lowercase letters indicate significant differences among genotypes at a given P level.

**Supplementary Figure 2** Box plots showing root length, root surface area and specific root length on the total root length of cotton genotypes grown under low or high P supply and inoculated with or without indigenous AM fungi. The groups (and bar patterns) were as follows: 1950-1960 (red), 1970-1990 (yellow), and 2000 to present (blue). We define those from “1950-1960” and “2000 to present” as the old and modern varieties, respectively. Boxes show first quartile, median and third quartile. Whiskers extend to the most extreme points within 1.5 × box lengths. The Wilcoxon test was carried out between the old and modern varieties or between with and without inoculation with indigenous AM fungi, and the *P* value is marked.


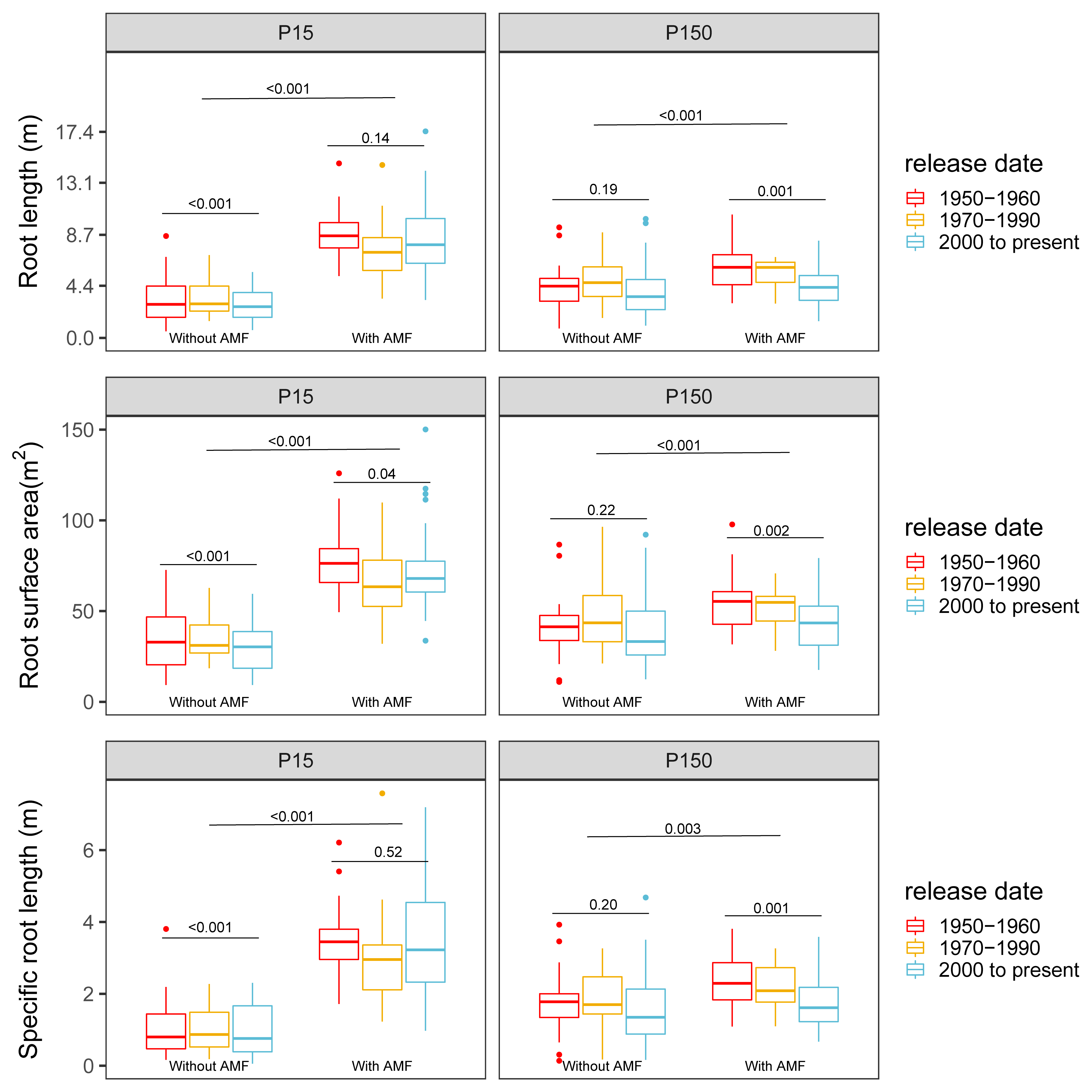

**Supplementary Figure 3** Phosphorus uptake efficiency (PUtE) of twenty-four varieties inoculated without or with AM fungi under low (A) or high P (B) supply. For each genotype, student’s *t* test is carried out between non-inoculation and inoculation treatments.
